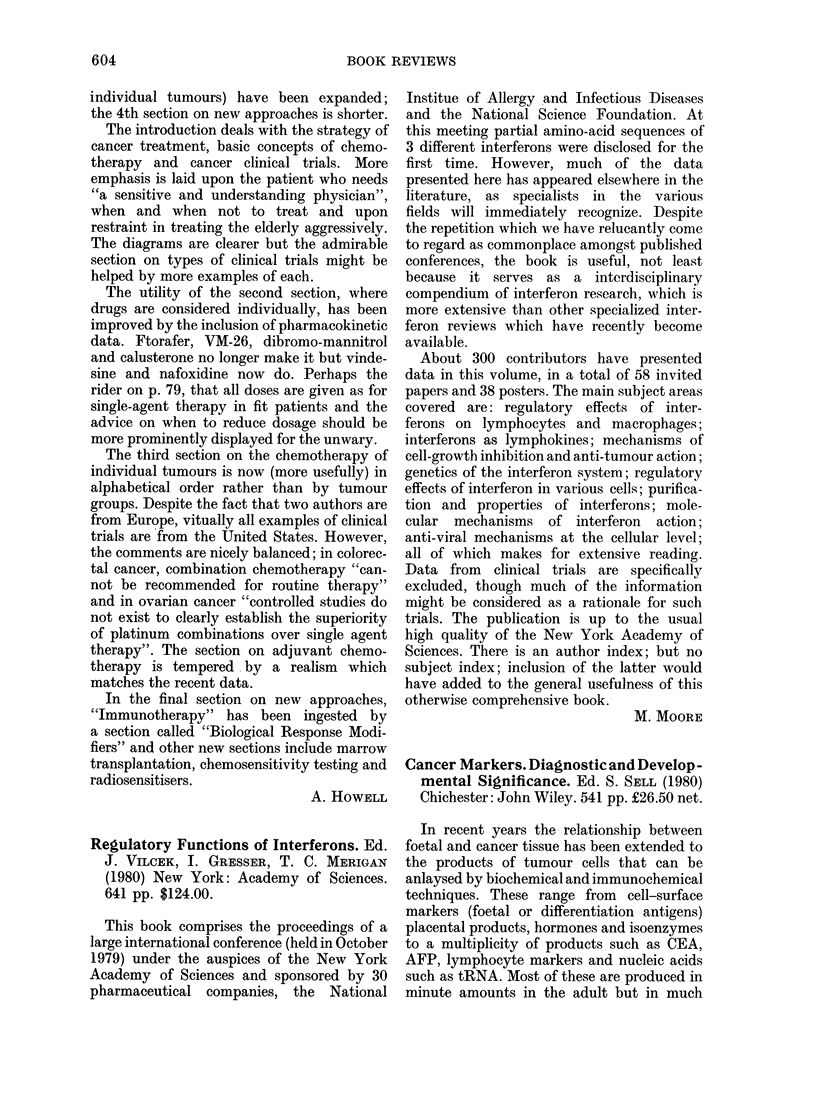# Regulatory Functions of Interferons

**Published:** 1981-10

**Authors:** M. Moore


**Regulatory Functions of Interferons.** Ed. J. Vilcek, I. Gresser, T. C. Merigan (1980) New York: Academy of Sciences. 641 pp. $124.00.

This book comprises the proceedings of a large international conference (held in October 1979) under the auspices of the New York Academy of Sciences and sponsored by 30 pharmaceutical companies, the National Institue of Allergy and Infectious Diseases and the National Science Foundation. At this meeting partial amino-acid sequences of 3 different interferons were disclosed for the first time. However, much of the data presented here has appeared elsewhere in the literature, as specialists in the various fields will immediately recognize. Despite the repetition which we have relucantly come to regard as commonplace amongst published conferences, the book is useful, not least because it serves as a interdisciplinary compendium of interferon research, which is more extensive than other specialized interferon reviews which have recently become available.

About 300 contributors have presented data in this volume, in a total of 58 invited papers and 38 posters. The main subject areas covered are: regulatory effects of interferons on lymphocytes and macrophages; interferons as lymphokines; mechanisms of cell-growth inhibition and anti-tumour action; genetics of the interferon system; regulatory effects of interferon in various cells; purification and properties of interferons; molecular mechanisms of interferon action; anti-viral mechanisms at the cellular level; all of which makes for extensive reading. Data from clinical trials are specifically excluded, though much of the information might be considered as a rationale for such trials. The publication is up to the usual high quality of the New York Academy of Sciences. There is an author index; but no subject index; inclusion of the latter would have added to the general usefulness of this otherwise comprehensive book.

M. Moore